# Burial workers’ perceptions of community resistance and support systems during an Ebola outbreak in the Eastern Democratic Republic of the Congo: a qualitative study

**DOI:** 10.1186/s13031-023-00521-0

**Published:** 2023-05-25

**Authors:** Hana Zwick, Marc Salama Asobee, Isabeaux Kennedy Mitton, Jennifer Headley, David E. Eagle

**Affiliations:** 1 Section of Infectious Diseases, Boston Medical Center, Boston, MA USA; 2 Christian Bilingual University of the Congo, Beni Town, Democratic Republic of Congo; 3 Results for Development, Washington, D.C. USA; 4 Duke Global Health Institute, Duke University, 310 Trent Dr, Durham, NC 27710 USA

**Keywords:** Ebola Virus Disease (EVD), Community health workers (CHW), Burial worker, Knowledge and misconceptions, Stigma, Belief systems, Barriers, Support systems, Democratic Republic of Congo (DRC), Qualitative

## Abstract

**Background:**

Community Health Workers (CHWs) provide vital services during disease outbreaks. Appropriate burials of those who died from an infectious disease outbreak is a critical CHW function to prevent infection and disease spread. During the 2018 Ebola Virus Disease (EVD) outbreak in Beni Town, North Kivu, Democratic Republic of the Congo, we sought to understand the levels of understanding, trust, and cooperation of the community in response to the outbreak, the barriers burial workers faced in their health work and its impact on local burial workers and other CHWs.

**Methods:**

12 EVD burial CHWs in Beni Town completed an hour-long qualitative in-depth interview on their experiences. They were recruited from a local counseling center. Interviews were recorded, transcribed and translated into English. A team of 3 researchers identified structural and emergent themes using applied thematic analysis.

**Results:**

Workers reported major misconceptions in the community surrounding the initiation of the outbreak. Community misconceptions were based on widespread governmental mistrust as well as a belief system that intertwines traditional and scientific understandings of the world. EVD burial workers identified violence directed at them and community misinformation as the two largest barriers to effectively carrying out their work. They named several important support systems including family and friends, personal relaxation techniques, and a local counseling center.

**Conclusions:**

As with other disease outbreaks globally, we found that government mistrust and religious beliefs strongly impacted community perceptions of the EVD outbreak. Previous studies have demonstrated clinic-based medical personnel are often the targets of violence. Our research shows that burial workers were also targeted and exposed to extreme levels of violence in their work. Along with their ability to effectively respond to the outbreak, violence has a negative impact on their own mental wellbeing. Burial workers found group counseling sessions to be an effective tool for managing the stress associated with their work. Further developing and testing of group-based interventions for this group is a priority for future research.

## Background

The Democratic Republic of Congo (DRC) has a long history with Ebola virus disease (EVD) outbreaks; the first known human outbreak occurred in 1976 and the most recent in 2022 [1, 2]. Effective public health response is hampered by large-scale problems including limited health system infrastructure, widespread poverty, large numbers of internally displaced persons and ongoing armed conflict [3, 4]. Social resistance, in the form of government mistrust, suspicion of outsiders, and related conspiracy theories are equally important factors that limit effective public health response [2–4]. Research has shown that social resistance during active EVD outbreaks is related to the persistence of misinformation about its origins, treatments, and cures [5].

Safe and effective burials are critically important during EVD outbreaks to prevent the spread of the disease, but often upend traditional burial practices, which can create significant community resistance and fuel the spread of misinformation [6]. Because the emotions surrounding the death of a loved one run high, understanding community misperceptions about the causes, origins treatment and management EVD is especially important for the protection, training and support of burial workers [7].

Misinformation is common during disease epidemics and is often directly linked to mistrust of the government. One study in the North Kivu region of the DRC found that in a recent outbreak, 12% of survey participants believed that the disease was entirely fabricated and 72% were unsatisfied or mistrustful of the healthcare response to the outbreak [8]. As a result of this mistrust and misunderstanding, health workers often bear the brunt of the public disbelief and anger, with their services refused, resources limited, and lives threatened [9–13]. Between August of 2018 and November of 2019, 386 attacks, 7 deaths and 77 injuries were recorded in DRC against healthcare workers (both international and residential) and the health system [14]. Research suggests that attacks on healthcare are significantly underreported [15]. Healthcare workers face significant personal risks and are often hampered in their ability to aid their communities [2, 3, 8]. This can lead to higher levels of Ebola transmission and an increased fatality rate, and also to serious negative physical and mental health effects on the workers themselves [5, 11, 16–18].

In the context of burial work, the religious beliefs and spiritual practices of local communities are important to understand [19]. Religious and spiritual beliefs shape how people respond to infectious disease outbreaks [20–23]. Community beliefs around the origins of Ebola often include supernatural elements, which underscore the important role that religious and metaphysical belief systems play in communities in the DRC [21]. Religious and spiritual beliefs cannot be easily separated from beliefs about health – in the context of an Ebola outbreak, they shape peoples’ perceptions of Ebola, its origins, the ways in which care is sought, and the kinds of public health measures people are willing to adopt [23–25]. Religious leaders often serve as a line of stable and trusted communication. In under-resourced settings, some people rely on traditional medicine and local religious healers for primary health information and care [21, 23, 26].

It is clear that EVD outbreaks have a significant negative effect on CHWs [11, 27]. With burial workers in particular, little research exists about their personal experiences, the barriers they face, and the support systems they rely on in order to do their important work. This study, one of the first done exclusively with burial workers, aimed to identify (1) the popular conceptions of the origins and causes of the Ebola outbreak; (2) major barriers EVD burial workers experienced in delivering care; and (3) the support systems that they relied upon to help overcome those barriers. We aimed to learn more about the community’s perception of Ebola through the eyes of the burial CHWs, who were experiencing the ramifications of those perceptions in their daily lives. Understanding the hesitancies and beliefs within the community allows burial workers and their supervisors to tailor their sensitization techniques and information, effectively and efficiently providing accurate information to the public and encouraging healthy burial practices. Therefore, a solid grasp on community perceptions and related CHW experiences is critical in understanding how to best support burial and other CHWs serving in contexts where there is both a disease outbreak and significant social and political instability.

## Methods

The study was conducted in Beni Town, North Kivu Province, DRC from April to July 2019. Beni Town was one of the epicenters of the 2018–2020 Ebola outbreak and reported 736 probable and confirmed cases. A total of 12 purposively selected EVD burial workers were identified and approached to participate in qualitative, in-depth interviews (IDIs) about their experiences working on the frontlines of an EVD outbreak.

Participants were recruited by leaders of group counseling sessions for Ebola workers offered by the Bethesda Counseling Center in Beni Town. Bethesda Counseling Center is a Christian counseling center affiliated with the Christian Bilingual University of the Congo, where the second author is a faculty member. Participants were offered the opportunity to participate, and if they consented, the interviewer collected the participant’s name and WhatsApp number to schedule a time to conduct the interview. The interviewers retained the participant’s information in the event of follow up questions related to the interview (up to a period of 12 months, after which this information was destroyed). This purposive sampling continued until the research team agreed that data saturation was likely reached [28]. All twelve workers who were approached initially accepted the invitation to participate in the study. Two participants later became unavailable due to personal or professional reasons, and were replaced by other purposively selected burial workers.

Participants were eager to be involved in the study based on their (a) willingness to make their challenging experiences known with the hope to raise awareness locally and globally, (b) desire to reflect about personal experience and find rest and renewal by sharing their stories with other people in addition to the supportive environment provided by the Bethesda Counseling Center, (c) readiness to find a voice in a hostile context where burial workers are seen as betrayers and a threat to the community. Participants were all male, reflecting the local culture and gender-specific job divisions. Women in this context are rarely, if ever, involved in burial work. These respondents represent a variety in terms of age, religion, and years of work experience, which expands the scope of our results, while remaining specific to the context of Beni Territory.

IDIs were conducted using a semi-structured guide in Swahili or French (participants’ preferred language) and lasted 60–90 min. Interviews were conducted in a location that was comfortable for each interviewee. The interview guide was developed through an iterative process between the Primary Investigator (PI) and staff at the Integrated Research Institute (IRI) at the Christian Bilingual University of the Congo (UCBC), the local research partner organization. Major topics covered in the guide included perceptions of the disease, delivering care, and personal impact. Interviewers were drawn from the staff of IRI and were trained in conducting interviews and protecting confidentiality. Interviewers were matched by gender with the respondents. Oral consent was obtained from respondents prior to beginning the interview. Interviews were recorded on digital recorders. Respondents received $US10 cash as a participation incentive. Interviewers transcribed the recordings. The site investigator at IRI then checked and cleaned the transcripts, redacted all potentially identifying information, and supervised a team of translators from the faculty of UCBC, who were all fluent in English, French and Swahili, to translate interviews into English. The translation team met weekly to discuss how to handle difficult translation issues, especially for medical terminology. Participants were assigned an identification number, which was used to identify their responses. After translation, the file linking the respondents to their identification numbers was destroyed. In all quotations that follow, pseudonyms are used throughout to protect participant confidentiality. All study procedures were approved by the Duke University Institutional Review Board. Local IRB oversight was not available.

Two qualitative analysts from the US based at Duke Global Health Institute reviewed all transcripts and generated an initial codebook of both structural and content codes based on the interview guide and participants’ responses. The analysis followed a thematic analysis approach, with analysts identifying key themes in text transformed into codes and applying data reduction techniques. [29] To further refine the initial codebook, each analyst wrote multiple cross-case memos to explore and identify emerging content codes. The analysts regularly met with a qualitative supervisor as well as with the PI and site PI to discuss codes and analysis summaries. Once the codebook was finalized, the two analysts simultaneously coded 3 of the 12 transcripts to compare coding application. Any instances of disagreements in codes were discussed and the analysts came to subjective agreement and revised codebook definitions as needed. After conducting inter-coder reliability on 3 transcripts and finding the two analysts consistently applying codes across the transcripts, the remaining 9 transcripts were split between the analysts and coded individually in NVivo 12. [30] After coding, coding reports were pulled and analysts wrote code-specific memos highlighting sub-themes, relationships between each other and relevancy to research aims. Data reduction tables were used to pull relevant frequencies for themes and sub-themes.

## Results

A total of 12 EVD burial workers were interviewed. We present the results from each aim as follows: Aim 1, where questions focused on the popular conceptions of the origins of the current EVD outbreak. Under this aim, we identified three major themes. We also recognized temporal shifts in people’s attitudes and also describe those under Aim 1. Aim 2 focused on responses to questions about the barriers workers faced doing their jobs; participant reports crystallized on 3 major barriers. Finally, we identified 5 key supports upon which burial workers relied to do their jobs and describe those under Aim 3.

### Aim 1: Determine the common/popular conceptions of the origins and causes of current Ebola outbreak in Beni Town

#### Theme 1: politics

The most common community-perceived cause of the Ebola outbreak, noted in responses to open-ended questions by almost all (11/12) interviewees, was politics. The transcripts reflected a widespread belief in Beni Town that members of the government of the DRC had either created or made up the disease in order to maintain power. One participant reported, *“The people of Beni said that Ebola does not exist. ‘Health workers deceive us, it’s politics.’” (Joel)*. Workers identified three common opinions among community members that explained why politicians would deliberately introduce Ebola into the area: voting restrictions, tribal persecution, and monetary gain.

The first suggestion was that the disease was a cover, giving the government an excuse to exclude the region from voting in an important election. In December of 2018, a presidential election took place in the DRC that removed the incumbent Joseph Kabila and elected opposition leader Félix Tshisekedi to the position [31]. Former President Kabila was referred to in a negative manner twice, and the December presidential election was specified once, referred to simply as ‘the election’ in other transcripts.

*“In the community, people say that the Ebola virus disease was imported by the government of former President Kabila, so as to prevent the holding of elections, to remain in power” – Kizito*

*“I can say that stories that people told me were basically about December 31 because three days before the elections it was declared that the population of Beni was not going to vote and people concluded that those same people created the epidemic to penalize the population.” – Felix*

The second political reason for the Ebola outbreak mentioned by interviewees was tribalism and the persecution of the Nande tribe specifically. One worker mentioned that the province of North Kivu, which includes Beni Town, is majority Nande. There is a belief that the people of Beni Town are being attacked because of their leader’s tribe.

*“It was said that the massacres failed, they have imported Ebola, that’s what people say. Even scholars told us that Ebola is a disease that was conceived by scientists and used by politicians to play their games, to show the leader from this region he is nothing, as he is Nande. Therefore, his people will be exterminated.” – Kizito*.

Finally, participants reported that there was a perception among community members that the government benefits financially from the epidemic. Non-governmental organizations (NGOs) sent aid and humanitarian assistance to Beni Town and the DRC to alleviate suffering caused by the violence and then the Ebola outbreak, and some community members perceived the relationship between the NGOs and the government to be opaque and corrupt [13].

*“It is said, for example, that if this epidemic recurs, it is because of the Congolese government. The government is making money from the epidemic, then foreigners bring us money.” – Bienfait*

These three mechanisms (voting exclusion, tribal persecution, and monetary gain) were the most commonly reported community conceptions of how the government may be using the Ebola outbreak to its advantage. These specific conceptions are based on underlying notions within the community. One such notion, brought up by five different interviewees, was that the timing of the outbreak intertwined with instability and violence in the country. They noted that as the outbreak came at a time when politically-motivated violence was sweeping the region, the two things could be perceived as tied together. Community members were often unable to disentangle the two, therefore transferring their blame on the government from the violence to the disease outbreak. A notion mentioned multiple times was that the violence was the first step taken by the government to meet their end goals, and when that was ineffective, officials turned to utilizing Ebola as a weapon.

*“Really the people of Beni unhappily learned about Ebola in Beni, Ebola arrived in a war period. They thought it was politics.” – Augustin*

#### Theme 2: the (Super)natural

When asked an open-ended question about community conceptions of Ebola, many interviewees brought up stories focused on spiritual or metaphysical origins. All 12 interviewees recognized that community members sometimes turned to traditional healers when they were trying to prevent or treat Ebola. They turned to traditional healers for a variety of reasons, including the lack of trust in the government health system and the belief that Ebola had supernatural origins requiring treatment that health facilities could not provide.

More than half of the participants stated that there are community members who believe in metaphysical origins of the disease. Of those who mentioned metaphysical origins, most interviewees directly mentioned witchcraft or evil spirits.

*Yes, some people turn to these imams, pastors, believing that they are possessed by evil spirits - Dieu-donné*

*On Ebola, people have told me that the origin of Ebola is that some people would have eaten cat meat, others say it is witchcraft. And it’s almost everyone who talks about witchcraft and politics. – Eleazor*

A few of those interviewees who referenced metaphysical origins specifically mentioned a cat as a central figure in stories about the origins of EVD, though the specifics differed. Joel related how some community members describe the owner of the cat as casting curses while Augustin referred to community rumors in which the cat was believed to be magical itself. Eleazor’s quote above simply mentions community members referencing eating cat meat as the disease origin.

*Some people say it comes from a cat. People ate the cat. The owner of the cat started to cast curses. – Joel*.

*There are rumors that people in Mangina ate a magic cat and the magic followed them – Augustin*.

Notably, five burial workers asserted that they themselves (unrelated to community conceptions) were aware that the cause is animals. Three mentioned bats, which much scientific evidence points to as an important reservoir of Ebola, a zoonotic disease [21, 32]. Given that they have received formal training to conduct their work, it is likely that EVD burial workers had more access to scientific information on EVD than the community at large.

*Far away, we know the origin of the Ebola virus. It’s said that it first appeared in Congo and was caused by a bat, which is the reservoir of the virus. – Felix*

#### Theme 3: human transmission

Interviewees reported that community members seemed to have a general understanding that human-to-human transmission of Ebola occurs, and this was often reflected in stories of individuals who brought the disease to different places.

*Others say that it came with one military person from Equator province to North Kivu. That’s the origin of the disease in Mangina. – Joel*

This was also shown through stories of prevention and treatment practices. Although these were not directly related to the causes of Ebola, the comments show an awareness of human-to-human transmission that offers a glimpse into the community’s understanding of how the disease moves, and therefore how it arrived in Beni Town.

*Apart from hand-washing, the population is abstaining when there is a case of death; they refrain from touching the body or sitting where there is a case of death. – Bienfait*.

Five burial workers clarified their own understanding that the disease was brought to Beni Town by a person, all with a specific individual, a wood cutter, in mind. This likely represents the information being provided by the Red Cross or another common authority, as the statements were very similar across all five interviewees.

*According to our training, Ebola is here in Beni and Mangina because of the wood cutter who came from Mbandaka and died in Mangina from Ebola virus disease. – Augustin*

*The epidemic is caused by a wood cutter who came from the equator province and died in Mangina. – David*

#### Other: understanding of Ebola over time

Seven interviewees stated that the community’s perceptions of Ebola and its causes had changed for the better over time. At the beginning, the community’s attitude towards the disease was characterized by uncertainty, suspicion, and skepticism. The controversy over the December election played a role in this uncertainty, blurring the lines between truth and reality.

*The reaction of the people of Beni was very bad because the people said that it is politics, a reason to delay the vote or it is a human sacrifice from the president of the Republic. – Eleazor*.

A lack of trust in the entities communicating the message about Ebola also shaped the initial conceptions of the community with cynicism and doubt.

*The people in Beni said that Ebola does not exist and that it is a phenomenon to make money and they add that if Ebola existed many people and animals would be dead. – Augustin*

Over time, however, opinions changed and community attitudes shifted. A few interviewees attributed this process specifically to sensitization and awareness-raising done by the burial workers themselves.

*Yes, there are changes. For example, in beginning, my neighborhood and people around me didn’t look at me positively. Even when our visitors from other provinces began to raise awareness, the community saw them badly. But today, the more we raise awareness in our community, they are convinced. We communicate very well. – Joel*

A couple interviewees ascribed the shift in understanding to the increase in deaths within families and social circles. Eventually, the evidence of the existence of a fatal disease became indisputable.

*There are people who start understanding that the epidemic exists because as one person from their family could be buried and the family keeps resisting. then the younger brother of the dead could fall sick and passed on. We have tangible proofs as it is our work. – Felix*.

However, a few interviewees confessed that there remained resistance to the reality of Ebola. One believed that the minority of the community members were fighting back against the response teams while another believed it was the majority. A third interviewee, Negasse, believed that although many people have not come to an understanding, progress is being made over time.

*Until today there are those who still resist. – Kizito*

*I think that many people have not yet realized the reality of Ebola but little by little they are beginning to understand that the disease exists. – Negasse*.

### Aim 2: major barriers that burial workers face in delivering appropriate care

#### Theme 1: violence towards burial workers

When asked what experiences impacted their work, all interviewees cited violence as a barrier to delivering appropriate care. All interviewees reported experiencing either physical assault and/or verbal assault when attempting to conduct safe burials in communities. Three-quarters of interviewees reported stones being thrown at them and/or their vehicles, experiencing verbal insults and physical assault, and at least one more interviewee experiencing verbal insults, but not physical assault. When interviewees were asked to describe the main difficulties or challenges burial workers encountered in Beni Town and in the deliverance of their care, several interviewees identified violence as the most significant barrier.

*When it comes to safe and dignified burial, sometimes we fight, people attack us even to the point where they want to beat us or put us in the tomb. But those who know that the disease exists, implore us; but those who do not know that the disease exists, even want to kill us at the graveyards. So they want to bury us alive in the place of the corpse. – Bienfait*.

Whether asked directly or raising concerns voluntarily, three-quarters of interviewees reported fears of being attacked for their work. As a result of experiencing violence, interviewees reported experiencing psychological concerns, such as trauma, mood and affect changes, and lack of sleep. When asked about how these experiences impact people’s work, interviewees responded affirmatively with statements such as,

*Yes, especially in my team … I can say that it is the trauma. – Joel*.

*There are times this fear gives me insomnia and nightmare (I dream for example of people who knock at my door or ask me for money) because the community says that we earn a lot of money.* – *Bienfait*.

*Our mood, when we are called by saying that this is a positive case, we also get emotions, and we wonder when this epidemic will end?* – *Joel*

#### Theme 2: Community’s mistrust and misinformation regarding EVD and caregivers’ work

Another reported barrier for burial workers to the delivery of appropriate care was community members’ mistrust and misinformation regarding EVD and burial workers’ work. Ten of the 12 interviewees mentioned that community members incorrectly believe burial workers are paid exorbitant amounts of money to do their work, as Dieu-donné expressed,

*People often say that we are the ones who receive a lot of money and yet we as volunteers do not have a contract, we are volunteers, and we can only have a small per diem so that we could eat well at home. – Dieu-donné*.

Interviewees also reported misinformation surrounding rumors that burial workers were harvesting organs and body parts to sell. These rumors in turn made community members mistrustful of burial workers when they would come to remove bodies or perform safe burials.

*And also in the community people say that the response teams work for Kabila as they look for some parts on the corpse body. So, when someone is dead, his sex and tongue are cut off. So, there parts of the body that are cut to sell somewhere. These are the rumors in the community. – Dieu-donné*.

Interviewees revealed how common and popular community conceptions of the origins and causes of the Ebola outbreak are founded in misinformation, leading to community members mistrusting burial workers. This mistrust in turn is manifested through violent acts against burial workers.

#### Theme 3: resource availability

While the research team initially hypothesized that a lack of resources would be a significant barrier for burial workers in conducting their work, when asked whether workers received adequate materials to conduct their work, all interviewees responded affirmatively.

*Interviewer: According to you, in your job or jobs, did you receive the necessary resources from you and your colleagues to help you work effectively?*

*Interviewee: Answer: yes, we have the chlorine, other medical combinations, spades … which helps us to prevent [infection]. – Felix*

### Aim 3: support systems available to burial workers

In each transcript, interviewees were asked questions regarding support systems available to burial workers. Interviewees identified five major sources of support. First, all interviewees mentioned the importance of their co-workers and the support of the Red Cross for their work. As Kizito reported,

*At home we are like brothers, so we take the other as our brothers and sisters, we love each other. When we are at the head quarters we play cards. When there was a difficulty and that one of us got sick, all of us went to support him, to see him. So the teams of SAFE AND DIGNIFIED BURIAL of the Red Cross are really well bonded, we collaborate, there is love between us. – Kizito*

All interviewees also mentioned the importance of family and friends for coping with the difficulties of their job. As Jean affirmed,

*I do not speak about problems or difficulties of my work because it is confidential. When it comes to support, I get support from my family and friends. My work did not affect my relationships. – Jean*

Bethesda Counseling Center, the recruitment site for this study, was also mentioned by all respondents. The group counseling was identified as vitally important,

Interviewer: *So, you were part of a support group at Bethesda Counseling Center, did participation in this program help you?*

*Interviewee: Yes, it helped us a lot; moreover the team wanted us to continue with it. What we did not know and that we learned was just how we can take care in case of difficulty. For example, when you are angry, when you remember what you have been taught, you take care of yourself. – Bienfait*

Most participants mentioned their faith or religion providing comfort and support, or even instructing the community to let burial workers do their jobs without harm For example, Eleazor stated,

*Indeed, my faith helped me to do my job more effectively. It has helped me to the extent that it gives me the freedom to do this work and encourages me. – Eleazor*

And finally, participants also recounted how they found personal relaxation and important way to manage the demands of their job. As David shared,

*Interviewer: what kinds of things do you do to relax?*

*Interviewee: I listen to music and look for a loved one to share stories. – David*

Most participants stated that through the various support systems, they were able to focus on relieving stress and finding a sense of community among other burial workers. The counseling center provided tools and techniques for remaining calm in stressful moments, as well as processing events after they happen. Many participants recount successfully using those techniques in a high-stress situation, and express gratitude to the counseling center for teaching them. One participant stated that psychologists were always available to the workers, allowing more immediate access to a critical piece of the support system.

Finding a sense of community was important to the participants. They often mentioned difficulties remaining in touch with family and friends due to the controversial and dangerous nature of their jobs, and also described feelings of distressing loneliness. Participants felt higher stress and sadness when they were alone, and through work, the counseling center, and religion they were able to build a community around them. With other workers, participants were able to watch movies, play games, and spend time together without focusing on the traumatic aspects of their work. Finally, the counseling center encouraged forgiveness. When workers were able to understand and forgive community members for any negative actions, one participant stated that they were able to connect and collaborate with the community.

## Discussion

This study was initiated to understand the experiences of EVD burial workers during a major outbreak. The main goals were to understand community conceptions of the origins and causes of the 2018–2020 EVD outbreak in Beni Town, DRC, as well as to identify barriers that burial CHWs face in responding to the outbreak and the support systems that sustained them. We solicited the perspectives of 12 burial workers who all participated in group counseling sessions at a local counseling center. In line with previous studies, our findings suggest that the community held misconceptions surrounding the initiation of the outbreak, based in widespread governmental mistrust as well as a belief system that intertwines traditional and scientific understandings of the world. Barriers that burial workers faced in delivering their services were identified, with the two most prevalent being violence and misinformation. Support systems noted by the participants include family and friends, personal relaxation techniques, and the Bethesda Counseling Center.

The two most common community perceptions of the origins of EVD in the DRC were politics and supernatural causes. The first stems from significant pre-existing mistrust in the national government, causing people to think that the government was using the outbreak to their advantage - either by entirely making up the disease, by creating and releasing it themselves, or by way of significant manipulation of the disease and the people. This is consistent with prior research in the DRC that emphasized a widespread lack of trust in the government. One 2019 cross-sectional study found that only 40.5% of respondents in Beni Town and Butembo (a neighboring region) trusted the national government to handle the EVD outbreak, and another found that 72% of those surveyed in Eastern DRC expressed dissatisfaction or mistrust in the EVD outbreak response from both the national and local authorities [8, 13]. These frustrations were echoed in a 2018 qualitative study where participants claimed that EVD allowed the ruling party to illegally extend its rule [33]. Our findings substantiate these past results, emphasizing sustained local mistrust in the government and the widespread perception that the government was manipulating the EVD outbreak to its advantage.

The second major community conception of the origin of the outbreak was supernatural causes, referenced by over half of interviewees. These causes were perceived to have manifested in different ways, including through a magic cat, witches, or evil spirits. These findings are consistent with a 2018 qualitative study in the DRC, which identified that participants “often endorsed the idea that the symptoms of Ebola come from witchcraft, curses, or bad luck” [33]. This does not give an idea of how saturated that conception is, only that it is prevalent across the country. Another study done in 2019 in the DRC specifically mentions a rumor about a witch casting curses that started the Ebola outbreak after her cat was eaten and found that 6% of their survey respondents endorsed supernatural origins of Ebola in general [21]. All of our participants mentioned traditional healers, a finding that is supported by other research, which found that when the origins are thought to be outside of the health system, individuals adopt preventative measures and treatments that are outside of the health system [33]. Our findings support prior studies that underscore that belief in supernatural origins of Ebola are significant in local communities in the DRC. These beliefs play an important role in how those communities respond to outbreaks, including to safe burial teams.

Over time, initial perceptions of the origins of Ebola seem to have shifted away from these two theories and towards a more biological understanding. Respondents in our study attributed this to awareness-raising activities as well as individuals watching family members pass away, with traditional healing methods making no difference. A 2018 commentary from Mangina, where Ebola is known to have originated before spreading to Beni Town, also emphasizes that the community showed a change in understanding and a decrease in active resistance against response teams due to a ‘multidimensional approach’ that included communication with the community, case management, safe and dignified burials, surveillance and contact tracing, and more. [9]. A 2019 case study from the Ebola outbreak in Sierra Leone also found that community perceptions were reshaped over the course of the epidemic, with change being mostly attributed to social contact with Ebola workers and personal experience with the disease [12]. This suggests that it is feasible to replace initial misconceptions with facts and evidence-based information.

There was an expectation that CHWs would cite lack of sufficient equipment and resources as a major barrier to their work – this has been identified as a major barrier in delivering adequate maternal care in the DRC [34] and was cited as a major barrier to delivering hygiene kits during a cholera outbreak [35]. Contrary to these expectations, workers reported being generally satisfied with the availability of supplies, but identified personal safety and community suspicion as the largest barrier to delivering effective care. Participants cited being attacked by individuals and groups as a barrier to delivering appropriate care. Incidents of assault included individuals throwing stones at caregivers and their vehicles, individuals or barricades blocking caregiver’s vehicles on roads, or individuals verbally threatening to attack and even kill the caregivers. Interviewees expressed they were fearful of being attacked due to their work, and that these incidents of verbal and physical assault caused many of them to experience trauma, mood changes, and lack of sleep. These violent attacks on burial CHWs not only physically prevented them from performing their work but placed a high psychological strain on burial CHWs individually and collectively. Reports of assaults on Ebola workers are similarly reported in a 2019 study exploring persistent Ebola transmission due to community resistance where participants stated health workers were injured by stones thrown at them [8]. Other reports cite the direct impact violence due to political instability has on CHWs’ work [2]; however, the impact of violence and the physical and physiological impact this has on CHWs is not as well documented as that against clinic staff. A study conducted during the 2013 Ebola outbreak in Sierra Leone, Guinea, and Liberia found that while health facilities were strongly distrusted, CHWs held a unique position by being associated with health facilities but having a closer connection with community members due to the nature of their work [36]. Our work serves to bridge the gap in knowledge that exists surrounding the unique position of CHWs and their experiences and relationships with community members. Several news reports [14, 37] have reported attacks on Ebola workers without a clear understanding of motive, but it is evident from our findings that Ebola workers are often intentional targets of violence.

A second barrier to care reported by participants is the community’s mistrust and misinformation of burial CHWs’ work. Participants from the study reported community members held inaccurate conceptions of their roles and responsibilities, where some community members believed burial CHWs were substantially financially compensated or that burial CHWs were harvesting and selling organs and body parts from deceased Ebola patients [6]. As mentioned previously, communities held misconceptions of the origins of EVD and expressed mistrust of and frustrations surrounding the outbreak response. Burial CHWs were not exempt from these community conceptions of EVD, which manifested in further misinformation and community-held rumors surrounding their work. Qualitative studies from 2018 to 2020 [9, 33] echoed our findings that community members from EVD-affected areas believed that community health workers were intentionally spreading Ebola in order to harvest and traffic organs. A 2020 study [33] reported that some patients mistrusted community health workers and sought alternative sources of care, rather than relying on the healthcare system. Our findings support results from these studies, detailing how barriers to Ebola response included community mistrust and misconceptions of the nature of safe burial work.

When participants from our study were asked how they managed their work, many interviewees cited support and help from a range of systems. Support systems included the Bethesda Counseling Center, faith and religion, personal relaxation, family and friends, and other co-workers/Red-Cross workers. Respondents’ affirmations that they sought out these systems to help manage their work demonstrates how important these systems are in supporting CHWs in their personal and professional wellbeing. Through their support systems, burial workers were able to learn relaxation techniques, process trauma, and find a sense of community. Their unity and togetherness were vital in maintaining their strength during a time of violence, unrest, and uncertainty. Though our study was not researching the impact of EVD on burial worker’s mental health, our findings demonstrate the major impact EVD and other epidemics or pandemics have on our interviewees and health care workers and how critical interventions that support the mental health of healthcare workers [11, 17].

Our study is subject to several limitations. Most significantly, we interviewed EVD safe burial workers about community conceptions rather than asking community members directly. This was done to give context and understanding to the barriers and support systems that impacted the workers during the outbreak but limits the conclusions that can be drawn from their responses. This could introduce bias into the first aim of the study specifically, as burial workers may have interacted with people who held a particular set of beliefs at a disproportionate level to the true distribution of beliefs, therefore misrepresenting their prevalence. A second limitation is the size of our sample, which is relatively small at 12 participants. While we reached saturation of major themes, the interviewees were all recruited from the same place and were all burial workers, increasing the risk of selection bias. Participants did vary in age, religion, and years of work experience, all serving to widen the scope of our results, but our results remain specific to Beni Territory, the 2018–2020 outbreak, and burial workers. Additionally, our participants were recruited from those taking part in group counseling sessions at the Bethesda Counseling Center, which may have led to bias in reporting the center as a support. In an attempt to help mitigate this potential bias, study interviewers were from the IRI and not associated with the counseling center. Additionally, our study does not include voices from burial CHWs who were not in group counseling sessions and their responses to barriers in conducting their work and the supports they rely on may differ from those who attended group counseling. Finally, our data was collected during an active Ebola outbreak. We were not able to capture the long-term impacts of the outbreak on workers. Longer-term support systems may not have been fully understood and enumerated. However, there is a benefit to conducting interviews in the midst of an outbreak.

Several recommendations emerged from our results, ranging from high-level concepts to specific community activities. First, this study underscores the importance of understanding how religious beliefs impact scientific understanding. Religious beliefs figured prominently in burial CHWs’ accounts of community resistance, a theme echoed in other research [26]. Awareness-raising activities were cited by our participants as a major combatant to misinformation and were attributed with a significant portion of the credit for shifting community perceptions over time, a finding backed up by other research [38, 39]. In places like the Eastern DRC, where State institutions are weak, religious communities often play a central role in responding to health crises as they are places where social trust tends to be high and can play an important role in health communication. [40]. Given the involvement of religious leaders following the death of a person, one promising avenue for intervention is to increase partnerships between public health authorities and religious leaders to challenge misconceptions of the origins of EVD and promote safe burial practices. Research suggests these partnerships are most effective when the perspective and concerns of religious leaders are not subordinated to health care provision [40]. Opening spaces for ongoing dialogue between leaders in the religious and public health spheres could provide stronger counter narratives to conspiracy theories and improve working conditions for EVD burial workers.

Second, our study demonstrates the importance of providing burial workers with safety and protection, both against armed political groups as well as violent community resistance. The plight of burial workers are not often the focus of research, yet they play a central role in pandemic response. Violence limits their ability to do their jobs and also exacts a significant psychological toll [16]. Our participants stated how the counseling center proved to be a critical support system for burial workers, helping them cope with stress and trauma related to the outbreak. Increasing the number of available programs at local counseling centers and reaching a higher percentage of workers could aid in mitigating the mental health impact of an outbreak on burial teams.

To implement these recommendations, further research is necessary. Our findings demonstrated the important role spiritual beliefs play in community perceptions of EVD. Religious belief systems interact with a community’s understanding of, and response to an outbreak such as Ebola. A 2019 mixed-methods study also found evidence that religious beliefs may shape transmission patterns, and combined with our findings, this supports a call for further research into the health-related impacts of an interwoven and complex belief system in the DRC [20, 41].

Finally, more research could be done to quantify the impact of local centers such as the Bethesda Counseling Center on CHW wellbeing. Our interviews touched on the overall impact of the center and the most helpful strategies it provided but did not go into detail about specific programs or activities. Deepened understandings of the nature of the supports found most helpful would allow for a targeted, strategic expansion of services to those in need. Better understanding who is not served by local service centers and why could also help in attempts to expand services to more burial workers where appropriate. Improving and/or expanding services would likely improve the mental resilience and overall well-being of individuals and communities going through outbreaks or other crises [11].

## Conclusions

In this study, we sought to understand the experience of burial CHWs during an EVD outbreak. Our findings support existing literature in suggesting that politics, religious beliefs and the government outbreak response are inextricably linked in the minds of communities, and that the relationship between them impacts community and individual responses to the outbreak. Misconceptions often fueled violence against burial workers, who personally experienced regular verbal and physical assaults as a direct result of their occupation. Support systems that our participants referenced included religion, families and friends and the local counseling center, which was reported to have a major impact on the resilience of the workers themselves. Our findings call for increased attention to the plight of burial workers during disease outbreaks and the need for (1) targeted responses to improve their working condition and (2) development of interventions that help reduce stigma, build social connections, and manage the consequences of direct and indirect trauma exposure.

## Data Availability

The datasets used and/or analyzed during the current study are available from the corresponding author on reasonable request.

## List of abbreviations

CHW: community health worker
DRC: Democratic Republic of the Congo
EVD: Ebola Virus Disease
IDI: in-depth interview
IRI: Integrated Research Institute
NGO: non-governmental organization
PI: Primary Investigator
UCBC: Christian Bilingual University of the Congo
